# An ethnography of chronic pain management in primary care: The social organization of physicians’ work in the midst of the opioid crisis

**DOI:** 10.1371/journal.pone.0215148

**Published:** 2019-05-01

**Authors:** Fiona Webster, Kathleen Rice, Joel Katz, Onil Bhattacharyya, Craig Dale, Ross Upshur

**Affiliations:** 1 Arthur and Sonia Labatt Family School of Nursing, Faculty of Health Sciences, Western University, London, ON, Canada; 2 Department of Family Medicine, McGill University, Montreal, QC, Canada; 3 Department of Psychology, York University, Toronto, ON, Canada; 4 Department of Anesthesia and Pain Management, Toronto General Hospital, Toronto, ON, Canada; 5 Department of Family and Community Medicine, University of Toronto, Toronto, ON, Canada; 6 Women’s College Research Institute, Toronto, ON, Canada; 7 Lawrence S. Bloomberg Faculty of Nursing, University of Toronto, Toronto, ON, Canada; 8 Clinical Public Health, Dalla Lana School of Public Health, University of Toronto, Toronto, ON, Canada; University of Colorado, Denver, UNITED STATES

## Abstract

**Background:**

This study reports on physicians’ experiences with chronic pain management. For over a decade prescription opioids have been a primary treatment for chronic pain in North America. However, the current opioid epidemic has complicated long-standing practices for chronic pain management which historically involved prescribing pain medication. Caring for patients with chronic pain occurs within a context in which a growing proportion of patients suffer from chronic rather than acute conditions alongside rising social inequities.

**Methods:**

Our team undertook an ethnographic approach known as institutional ethnography in the province of Ontario, Canada in order to explore the social organization of chronic pain management from the standpoint of primary care physicians. This paper reports on a subset of this study data, specifically interviews with 19 primary care clinicians and 8 nurses supplemented by 40 hours of observations. The clinicians in our sample were largely primary care physicians and nurses working in urban, rural and Northern settings.

**Findings:**

In their reflections on providing care for patients with chronic pain, many providers describe being most challenged by the work involved in helping patients who also struggled with poverty, mental health and addiction. These frustrations were often complicated by concerns that they could lose their license for inappropriate prescribing, thus shifting their work from providing treatment and care to policing their patients for malingering and opioid abuse.

**Interpretation:**

Our findings show that care providers find the treatment of patients with chronic pain–especially those patients also experiencing poverty–to be challenging at best, and at worst frustrating and overwhelming. In many instances, their narratives suggested experiences of depersonalization, loss of job satisfaction and emotional exhaustion in relation to providing care for these patients, key dimensions of burnout. In essence, the work that they performed in relation to their patients’ social rather than medical needs seems to contribute to these experiences. Their experiences were further exacerbated by the fact that restricting and reducing opioid dosing in patients with chronic pain has become a major focus of care provision.

## Background

Chronic pain is a significant health concern globally, affecting an estimated 20 percent of adults worldwide [1], and is among the most prevalent chronic conditions in Canada [2]. In the province of Ontario–the site of this study–it is the most prevalent chronic condition [3], especially among those with multimorbidity [4]. Chronic pain also disproportionately affects individuals with low SES [3]. This means that clinicians regularly engage with the constraints and challenges experienced by patients who struggle to cope with pain and other chronic conditions in conditions of poverty and marginalization. These circumstances were impetus to ground our ethnographic study in the standpoint of clinicians in primary care, as little is known about the actual *work* that is involved in providing care for these patients. An empirical understanding of the materiality of physicians’ work is crucial for understanding–and ultimately addressing–these evidently socio-medical conditions.

Like other chronic disease sufferers, patients with chronic pain have been found to use more health services than the average Ontarian, and to incur higher medical costs overall [5]. Yet despite the pervasiveness and expense of this condition, chronic pain is often poorly-managed [6,7], and chronic pain sufferers frequently struggle with depression, anxiety, and poor quality of life [8,9]. Indeed, Tang and Crane [10] report that risk of suicide doubles for chronic pain patients as compared with people without chronic pain. Furthermore, widespread incidence of chronic pain dovetails with a prescription opioid crisis that is currently at crisis levels in Canada [11]. While data linking prescription opioid use and mortality death to specific demographic indicators is extremely limited [12], data from British Columbia, Canada, indicates that prescription opioid use is four times higher among those of lower socio-economic status (SES) [13], and recent research from California [14] suggests that prescription opioid death rates are highest among those with the lowest SES.

Furthermore, evidence suggests that more broadly, chronic disease sufferers and high-cost health system users are disproportionately of lower SES [15,16]. For instance, the authors of a recent Ontario health record database study found that residency in low income neighbourhoods strongly correlates with becoming a high cost user of the health care system overall, and conclude that “addressing social determinants of health, such as food and housing security, may be important components of interventions aiming to improve health outcomes and reduce costs” [15]. This is unsurprising, given that in Ontario, as elsewhere, the burden of disease increases as SES decreases [17, 18], yet this well-known correlation is relatively muted in the literature.

Our current analysis focuses on physicians’ and other primary care providers’ reflections on their experiences of providing care to patients with chronic pain. While evidence suggests that the care that is provided to these patients often fails to meet patients’ hopes and expectations for pain relief and recovery [19, 20], our approach is not designed to evaluate the quality of care that our study participants provide. Rather, by turning a sociological eye to the challenges of providing chronic pain care, we are able to identify how the structure of the health care system and parameters of primary care as a scope of professional practice do a profound disservice to patients and care providers. Our study was carried out in Canada, but resonance between our findings and research from other contexts suggests that our findings are applicable to a range of healthcare settings.

## Methodology

This article draws on data from an ongoing institutional ethnography [21] of the coordination of care for chronic non-cancer pain in Canada. Developed by sociologist Dorothy Smith, institutional ethnography (IE) is an approach to research rather than a set of research methods. We have described this approach and how we have used it in our work previously [22]. IE uses people’s everyday work problems as the starting point for an exploration of the often-invisible social relations that scaffold and orient experiences [23, 24]. Smith explains ruling relations as being “that extraordinary yet ordinary complex of relations that are textually mediated, that connect us across space and time and organize our everyday lives” [24].

People’s everyday lives are sites of interface between individuals and a vast network of institutional relations, discourses, and work processes. The object of interest in institutional ethnographic research is that interface between embodied individuals and institutional relations [21]. IE emphasizes people’s work, particularly activities often not included in formal definitions of work (e.g. filling out forms), and how it is coordinated–often through text—with that of others. Texts refer to any document that has a fixed and replicable character (such as a policy, care pathway or as in this study, a set of guidelines). Texts are “activated” when they are read, completed or filled in (i.e. discharge forms). Therefore we explicitly trace any texts that participants mention in their descriptions of how they accomplish the work they perform. Our guiding research question was, “How do primary care physicians describe the work they do in caring for patients with complex chronic conditions?”.

Our interviews were supplemented with observations at four sites. These interviews followed an open-ended interview guide that was piloted prior to commencement of the study to ensure flow of topic areas. Following IE’s emphasis on what Smith describes as “work knowledge”, interview questions were organized around eliciting details about the work that clinicians performed in carrying out care for patients they deemed to be complex [25]. We asked explicitly for examples they could share of actual cases as a strategy to counter professionally authorized ways of speaking that were often abstract rather than experiential and thus descriptively empty [26]. Sampling in an IE study is purposive but does not follow standard strategies such as maximum variation for example [23]. Our team identified research sites, informants and texts as we proceeded through the process of inquiry [25]. Participants were recruited from across Ontario Canada and included clinicians working in large urban centres, small cities, and remote Northern communities. Interviewees were recruited via a scripted email that was approved by the research ethics boards of both our university and several academic hospitals where study-related observations were carried out. No participants declined to participate, nor did anyone withdraw from the study. Formal written consent was obtained prior to all interviews. Interviews ranged in duration from between 30 and 90 minutes, depending on the amount of detail that the interviewees chose to share. We collected a total of 61 formal, semi-structured interviews, encompassing care providers at all career stages. The findings from this study report from interviews with 19 primary care physicians and 8 primary care nurses.

Rigorous ethnographic research combines interview and observational data to reach a deeper understanding of how forms of knowledge, rationality, and experience are sustained, produced, and contested through everyday practices and institutions [25]. By observing everyday work practices alongside formal interview narratives, this approach exposes connections between phenomena that may not initially appear linked [27, 28]. For this reason, we collected roughly 40 hours of observational data in clinical settings by shadowing primary care physicians’ daily work in caring for complex patients. The observer, KR, took “scratch notes” that were written into more detailed fieldnotes immediately following the observation, and were typed up into more in-depth fieldnotes within a 24 hour period. These observations were complemented by ad hoc interviews the observer conducted in the field, the purpose of which was generally to gain clarification or insight into an observed event. For observations of clinical appointments, formal written consent was obtained from the observed primary care provider, the patient, and from incidentally-present healthcare personnel. Patients were consented by their care provider and not by the observer. That being said, our team understands consent as an ongoing process. In addition to formal consent, KR remained sensitive to any non-verbal clues that her presence might be unwelcome.

The procedural steps involved in analyzing data in an IE study are similar to those practiced in many other qualitative research approaches. Data is transcribed and coded so that it can be analyzed. Codes identify features of the data that are pertinent to the research questions and organize data into more concise ideas that can be eventually grouped into topics. They are often recurrent keywords or concepts that are supported by interview data (i.e. quotes). Codes reflect our analytic interest in explicating how the work that is performed by an actor in one situation (locally) is coordinated extra-locally. Through interviews, the IE researcher identifies a series of texts and discourses that are present in the language of participants as they describe their everyday work practices.

The first several transcripts and field notes were inductively coded by FW and KR independently; the researchers then met to compare their codes and achieve consensus on items to be included in a coding framework which was then applied by KR to the remaining interviews. Coding by multiple team members is a useful way of developing reflexivity rather than a tool to confirm the ‘truth’ of the data. Reflexivity used in this way refers to the process by which we critically examine our own assumptions about the world. Through comparison of our developing understanding of the transcript data, each member of the coding team has an opportunity to check their own assumptions and ideas. At the same time FW and KR de-briefed weekly to discuss each new interview. Larger team meetings were held at several key points during which we discussed and reflected on our emergent understanding of the data. These larger meetings were led by FW, an experienced sociologist and IE researcher, and included a multidisciplinary research team that comprised a post-doctoral fellow who held a PhD in medical anthropology [KR], a PhD pain psychologist [JC], two academic primary care physicians [OB, RU]. Several of these meetings were joined by an IE trained nurse [CD], a medical student [JER], and a PhD candidate working in health psychology [EO]. Data analysis was an interactive, inductive, and collaborative process that involved identifying emergent themes and theorizing the implications of this for our broader research topic. Nvivo 10 software was used for storage and organization of data.

## Findings

Our findings are organized around physicians’ experiences providing care to chronic pain patient, and are presented in a narrative style that demonstrates care providers’ changing role vis a vis care provision, the frustrations they experience in trying to effectively provide care for patients that they find challenging, and their struggles to effectively respond to situations that they feel helpless to address. We have drawn on the many examples in which physicians describe having to manage issues of poverty and marginalization in their work rather than strictly patient’s health concerns. We also note in their stories many examples of loss of job satisfaction, emotional exhaustion and, at the extreme end of the continuum, depersonalization.

We begin this section by sharing an extended account in which a physician discusses a patient who undoubtedly faces far more barriers to health than do many Canadians. However, similar examples featured regularly among the interviews that we collected with health care providers working in primary care and warrant including this account. This particular narrative highlights that while our research aims to examine the current context and work of providing care for patients with chronic non-cancer pain in the province, it necessarily also entails investigating the relationship between pain and poverty, as well as the challenges posed by socio-economic status (SES) differentials between doctors and patients.

*The one that’s just off the forefront of my head is a client who has a very significant personality disorder*, *so antisocial*, *has a lot of anger based on just long-standing childhood trauma*, *and has a lot of other comorbidities*, *the most recent being an amputation to his leg because of recurrent infections because of his diabetes and also hep C*. *So he was someone who had lots of pain*, *and a lot of it was due to the infections in his leg*. *… He’s in a wheelchair*, *he doesn’t really know how to use it*, *he’s on Suboxone*, *which is a medication that he was getting for both [narcotic] addiction and pain but it’s not enough for his pain at this point so he actually does need pain medication*. *But he can’t leave his apartment because there’s stairs*, *and … the washroom is downstairs … and it’s a mess*. *I mean*, *we’re trying really hard to get him into accessible housing as a priority*, *but it doesn’t seem to be happening*. *So I’m spending a lot of time advocating for him … But that’s an example of me putting hours and hours into helping a client who has lots of other medical issues going on*, *and I would love to be able to focus on that*, *but because he doesn’t have housing that’s appropriate that’s taking over the forefront of my energy (Interview 9*, *Family Physician)*

In this paper we suggest that aspects of poverty, such as those described in the example above, are a ubiquitous feature of primary care provision, and this is likely intensifying as a growing proportion of patients suffer from chronic rather than acute conditions alongside rising social inequity. This was regularly documented in care providers’ narratives, and was and confirmed observational fieldnotes such as “*the patient is quiet*, *morose*, *and unstylishly dressed in drab clothing*, *with holes in one shoe*. *Rather than a winter coat he had on three layers of sweatshirts under a light jacket*. *(…) The family physician asks him about his housing situation*, *recalling that at his last appointment the patient was concerned about losing his apartment*” (observational fieldnote 8).

Although many patients clearly struggled with poverty and marginalization, in clinical contexts patient care takes place at the level of the individual, honing in on patient behavior rather than the social inequities that inform the circumstances of people’s lives. In the extended quote above, for example, the physician describes a patient living in substandard housing and refers to both “childhood trauma” and “personality disorder” in the same sentence. While the reference to childhood trauma would suggest that the patient’s behavior is shaped by experiences beyond their control–and while some personality disorders are associated with childhood trauma–the socio-cognitive nature of both personality disorders and non-physical trauma challenge the parameters of what is typically understood as biomedical pathology. Accordingly, physicians are more likely to view people as being responsible for having personality disorders (because they are enduring character traits) as opposed to, for instance, schizophrenia, which they recognize as a disease.

Many of the participants we interviewed described a disjuncture between patients’ hopes and expectations for pain management and the reality of what physicians can provide in way of treatment, especially in the current climate in which they are under pressure to restrict opioid prescriptions, the historical mainstay of treatment for patients with chronic pain. Most primary care physicians expressed sentiments such as the following, in which the physician links the fact that the “patient is suffering” with their lack of effective treatment and ongoing pressure to not prescribe opioids.

*Well*, *lots of them are difficult because the patient is suffering and you don’t have effective treatment for them … It can also be difficult if they also have expectations of things that you’re going to do that either you don’t think is effective for them*, *or there’s not an evidence base for*. *I suppose a specific instance in chronic pain is use of opiates*, *where you’re trying to judge that and it’s difficult to know whether the person is drug-seeking or whether they’re needing it for their pain (Interview 7*, *family physician/clinician scientist)*.

Several physicians alluded to the work of trying to determine if “the person is drug-seeking”, as in the excerpt above, but were often vague about specifically how this was accomplished. Especially for physicians who were trained in an era when prescription opioids were the gold standard for chronic pain treatment, such anxieties were further complicated by a lack of support in helping their patients’ transition away from prescription opioids. This conundrum is summarized by the following family doctor:

*They’ve heard [that high doses of opioids are risky]*, *but they don’t know what to do*. *So let’s say they’re prescribing a high dose to a patient*, *and they’re worried about it*, *so they say to the patient*, *“Jeez*, *maybe we should taper*,*” and the patient says*, *“But I got terrible pain*, *and if you taper me my pain will be even worse*. *I need this drug*. *How could you do this to me*!?*” And the doctor has never been trained in how to say no to a patient (Interview 8*, *family physician)*.

Saying no to patients as an aspect of clinical work was one of many informal activities that physicians in our study referenced as being particularly difficult to enact. Physicians also cited the difficulty of testing, measuring, or accurately evaluating a patient’s pain in the absence of any clear bio-markers. In the account below the physician describes how challenging it is to ensure she does not “inappropriately prescribe narcotics,” especially when she struggles to trust “what the patient is telling”. She does not find the work of medicine “exhausting” but rather the efforts that are spent “sorting out organic gain from secondary gain”. This adjudicating of the legitimacy of patient’s requests for pain medication is a growing area of focus for most providers, as she describes:

*The one thing I still struggle with all the time is just the subjectivity again of chronic pain*. *I think it’s just because it’s more subjective than objective*, *so it can be challenging in that perspective*. *And*, *especially with our population that we deal with because we deal with many high-risk patients*, *sorting out organic pain from secondary gain from other issues or pain*, *is something that’s manifesting as pain but is actually depression or something else*, *it’s exhausting*. *…And*, *again in [this city] we have a really high diversion rate of our medications*. *We’re super high as far as the province goes for narcotic prescriptions*, *so that’s just always looming in the back of your head*. *You don’t want to be inappropriately prescribing narcotics (Interview 17*, *family physician)*.

Frustrations about the subjective nature of pain were complicated by concerns some physicians had about malingering patients, and patients who might abuse opioids. These concerns sometimes stemmed from fears of harming the patient through overdose or prescribing drugs that might end up on the street, a fear the following physician describes as “an awful dilemma”,

*Patients on very high doses of narcotics]*, *these are mistakes* … *it’s not even a mistake*. *You [the family physician] are in between a rock and a hard place with this stuff*. *You are a compassionate individual who doesn’t want to leave your patients in pain*, *you’re also aware that if you continue down this road you run the risk of killing your patient*. *What an awful dilemma to be in (Interview 6*, *family physician with specialization in addiction medicine)*.

And similarly, in the following account the physician describes “lying awake at night” worrying about opioid abuse and opioid prescriptions:

*So I think opioids–like*, *opioid abuse and opioid prescription–is a really*, *really big issue*. *You know*, *definitely I lie awake at night thinking about it*. *Like*, *it’s one of those things that I think is a huge problem*, *and particularly because it’s very difficult to know what to do*, *right*? *(…) [For example*, *I have this new patient]*, *he even asked me*, *the first thing he wanted*, *he said*, *“Okay*, *well*, *how many appointments is it going to take until we get up to the dose that I was on*?*” …and he knows the drill*, *right*, *he knows what is needed*. *And so it’s hard when he says*, *you know*, *“Opioids are not my drug of choice*, *like*, *I always did crack*,*” and so you’re kind of simultaneously trying to build a rapport with a new patient and tell them that you trust and you believe them and you want them to come to you*, *while also denying them something that they want and invalidating them and* …*so it’s tricky (Interview 11*, *family physician)*.

Descriptions of these “tricky” aspects of providing care were common among physicians in our study, and featured in a number of observations as well. It gives rise to a peculiar tension between the work of trying to “build rapport” with patients and communicate trust while simultaneously “invalidating them”. In addition, in these examples the physicians begin to describe the extraordinary work performed by patients who “know the drill” in terms of how they must accommodate the various requirements of different physicians in order to try and get what they believe they need for pain relief.

Concerns about opioid misuse were often ascribed to The College of Physicians and Surgeons (CPSO) Opioid Strategy, recently launched in response to the 2017 Canadian Guideline for Opioids for Chronic Non-Cancer Pain [29]. This seemed particularly acute among younger physicians, whose medical training had emphasized extreme caution around prescribing opioids and had instilled fears of losing one’s license should they prescribe inappropriately. This concern is clearly articulated by the following family doctor:

*I’m sort of afraid to [prescribe opioids]*. *Just the medical-legal aspects of opioids*. *You always hear about prescribers losing their licenses as a result of prescribing opioids and them being used in an inappropriate manner*. *It happened to one attending in my hometown (…) he had to start independent practice*, *like in a walk-in*, *and there are all these restrictions on his license and it was in the paper*. *I don’t want to be one of the people who ends up in the back of the CPSO [College of Physicians and Surgeons of Ontario] magazines or anything like that (Interview 58*, *family medicine resident)*.

Although such concerns were shared by many physicians, their narratives also showed they are hamstrung by the lack of supports for opioid therapies, the limited alternatives to opioids, and apprehensions about the risks that opioids can entail. The following observational fieldnote extract illustrates this challenge:

[Young rural family physician] indicated that he would be doing paperwork in the evening, and that some of it had to do with prescription opioids. I asked him what his “stance” is on opioids, clarifying that we’ve encountered a range of perspectives on the appropriateness of prescribing opioids and that some physicians seem uncomfortable prescribing them at all. This is his response, as recorded by hand: *I don’t agree with not prescribing at all*. *As I understand it*, *pain is subjective*. *I can’t measure it in any way*. *But it’s real*, *and we can do something to reduce it*. *I think most docs who don’t want to prescribe don’t want to because it’s a ton of work managing someone on opioids*, *and it’s really challenging to get them off of it*. *But we don’t have the support for that*, *and it takes a huge amount of time*. *It would take a huge amount of my time*. *And I didn’t go into med school to become a babysitter*. *(Observational fieldnote 2)*.

This physician’s statement that he “didn’t go into medical school to become a babysitter” further highlights how the work physicians are now being asked to perform is not exclusively medical and thus introduces both a type and volume of work for which they were not trained or prepared and often do not want. For example, it is worth emphasizing that this physician equates the work associated with managing someone on opioids as “babysitting” rather than caring. We noted that similar concerns about opioid misuse were even more ubiquitous and explicit with patients of low SES:

*I constantly struggle on wanting desperately to believe the patients of their pain*, *but having that fear that it’s being diverted*. *Medications are being diverted or not used appropriately all the time*. *So*, *the subjectivity of it I find I struggle with all the time*. *And*, *again when I graduated residency it was everyone is innocent until proven guilty type thing*. *But*, *I feel in our high-risk clinic almost it’s guilty until proven innocent*, *and that saddens me as a physician (Interview 17*, *family physician)*.

In summary, patients with chronic pain have largely been treated with opioid-based treatments that have led to escalating addiction and death, further burdening these patients, and leading their physicians to feel often “sad” and overwhelmed in providing care for them.

As demonstrated in the excerpt at the beginning of this article, the theme of poverty and marginalization featured prominently in the interviews we conducted. Poverty was presented as a powerful barrier to providing optimal care to patients with chronic pain, and many providers noted that disadvantaged patients were heavily represented among their patients with chronic pain.

The following example provides a rich description of the kinds of patients that frequently feature in clinician’s narratives about chronic pain patients. In the following account, the challenges posed by low SES are compounded by the limited services available outside major urban centres:

[*Patient has] long-term lower finances in the family*, *has never had a job with proper benefits*, *is unable to work because of his pain*, *has diabetes*, *can’t pay for his medications because of not having benefits*, *is awaiting surgery which probably will not happen for another year for his back because of our lack of specialists here in town and lack of available surgeons*. *Basically he’s [now] in a state where we can start helping*. *But*, *in order to try to actually get him to a place where he’s going to be healthy is probably going to take at least a year and a half or two years by the time we can get some more benefit programs in place for him …*. *We’ve got our community health workers and our systems in place to help him access drug benefits*. *We can help him with his financial benefits and we can hopefully try to get him in for the surgery as soon as possible*, *and then he’s going to have the recovery from that*. *But*, *he’s so de-conditioned at this point and his health with the diabetes is so poor*, *we worry about what his recovery from the surgery is going to be like*. *So*, *yeah*, *very complex*, *complex client*. *Very*, *very nice man*, *very patient with the whole system*, *but obviously very frustrated with it too (Interview 18*, *family practice nurse)*.

Again, the primary care provider, in this instance a nurse, describes work that falls outside the medical domain, such as obtaining health benefits and other financial benefits for this particular patient. Everywhere, but especially in semi-rural or remote communities, it was clear that most care providers were aware of the limitations that poverty posed in terms of the care that these patients could access:

*The idea that there’s any group of medications that will*, *you know*, *cure chronic pain or*, *you know*, *make a big difference*, *well*, *often it does but lots of times it really simply isn’t as good as our management of acute pain*. *So doctors*, *we need to give patients access to*, *you know*, *self-management*, *to cognitive therapies*, *to exercise*, *etc*., *etc*. *The problem is that there’s no one available to do that*, *or they’re only available for people with a lot of money (Interview 8*, *family physician*).

Without necessary services and supports, care providers were mostly able to recommend voluntary, elective lifestyle modifications. Some of these, such as massage, are often not covered under the government-funded universal health care system or even available in many communities. Our study also indicates that although patients may be encouraged to take up lifestyle modifications as a means of improving their health, they often do not manage to do so. This is apparent from the following observational fieldnote extract:

*[The observed appointment] was heavily focused on the patient’s lifestyle*. *They had a long talk about getting out of the house*, *and it became clear that the patient only goes out for essential errands such as buying groceries and filling prescriptions*. *The physician encouraged the patient to try to find other reasons to leave the house*, *and asked the patient if he had anyone he could meet up with socially*. *The patient said that there are a few guys he could call*, *but that they usually want to meet at bars and this is a “trigger” for him*. *In the ensuing dialogue it became clear that the patient struggles with addiction*, *and he admitted to using “a bit” in the past couple months* (Observational fieldnote 8).

Moreover, with chronic pain patients in particular, patients’ reluctance or inability to make lifestyle changes, combined with a strong patient preference for medical forms of pain relief, was a common challenge for clinicians and one that often leaves them unable to “go home at the end of the day and feel satisfied” in the care they provided.

This sort of frustration features in this abridged interview excerpt, in which a family doctor describes a patient with whom she is having a “hard time”:

*One of the ones that I’ve had a hard time with he’s young*, *probably 35*, *very*, *very overweight*. *He’s got a history of chronic low back pain*, *he has had a MRI showing some nerve root impingement*, *he’s been seen by a couple of neurosurgeons*, *and has been recommended to get surgery*. *He refuses because he’s too scared to have the surgery*. *He’s been on Percocet for a number of years*. *The issue with him was that he was just so paranoid of the health care system that he just wouldn’t even consider the other options [besides Percocet]*. *…So I’m really trying to do some self-management with him*, *because obviously his obesity is contributing to his pain*, *and there’s just no getting him motivated to move around (*..*) So yeah*, *it’s been difficult with him because there’s a huge wall*, *he’s just*, *like*, *no*, *that’s it*, *I just want this [Percocet]*, *I’m done*, *I’m not going to do anything else*. *I have a hard time with that (Interview 26*, *family physician)*.

Perhaps due to such frustrations related to the “huge wall” physicians perceive between themselves and patients, several interviewees were dismissive of some patients with chronic pain. However, most recognized that while lifestyle-related changes are indeed helpful for people with chronic pain, it is not realistic to expect most people to make those changes without access to services not currently funded, especially so if they are living in poverty.

## Discussion

In keeping with IE’s emphasis on an expanded definition of work, our findings demonstrate the shifting nature of the work performed by care providers causes them frustration, exhaustion and compromised job satisfaction. These are key characteristics of what has been termed physician burnout [30]. In the literature, burnout is often described as an individual phenomenon and one that is anecdotally on the rise due to stress although inconsistently defined. However, the empirical experiences underpinning this stress are rarely described. While we do not diagnose “burnout” in our physician participants, we take note of the many examples of experiences consistent with this term that they describe in their accounts.

Most often these responses were described in relation to a changing work environment in which providing care now frequently revolves around restricting and reducing opioid dosing in patients with chronic pain. The College of Physicians and Surgeons (CPSO) launched an Opioid Strategy [31] in direct response to the 2017 Canadian Guideline for Opioids for Chronic Non-Cancer Pain [29]. In it the authors described the following:

The College will continue to work with the Ministry of Health as necessary to identify levels of opioid prescribing and investigate prescribing practices that may be harmful to patients. The College is aware that while investigations may identify instances of risk of harm to patients when opioid prescribing is continued, there is also a very real risk of harm to patients when opioid prescribing is discontinued. Understanding and questioning prescribing practices is not intended to discourage appropriate opioid prescribing and we are urging physicians to not suddenly cease prescribing to patients currently on opioid therapy.

These guidelines are vague, with little direction around when and *how* specifically opioids should be discontinued or not. Yet the emphasis on “investigations” renders clear that oversight will be enacted in relation to physicians prescribing practices and that penalties may be imposed. The concerns with appropriateness of opioid use raise issues regarding the fundamental justification of medicine in treating chronic pain as well as patient poverty. It seems that in the context of opiates and chronic pain, concerns about the limits of clinician judgment and worries about legitimacy are creating untenable issues for clinicians.

Furthermore, even when patients with chronic pain have straightforward medical problems, our findings show that primary care providers are often unable to effectively facilitate these treatments because their patients struggle with more pressing and immediate concerns, such as housing [32, 33]. The prevalence of poverty among chronic pain patients has implications for the work that physicians are called upon to perform, for patient outcomes and also for physician well-being. This informal work includes navigating the divide between differing socio-economic backgrounds between care providers and patients, work performed by both providers and patients.

Evidence indicates that health care providers rarely share the same socio-economic background as such patients [34]. This is especially so for physicians, who tend overwhelmingly to be from affluent social strata [35–38]. Often these differences have been related to bias on the part of physicians [39–42], or discussed in terms of effective communication between physicians and patients [43–46]. Rarely are these phenomena situated as the product of institutionally produced inequities rather than individual biases. Our findings show that most care providers are well aware that they are unable to meet the many needs of patients with low SES. The physicians in our study described being frustrated, worried and distressed by their inability to address their patients’ most pressing needs. Yet training does not currently exist to help them manage these issues. As noted by David Hilfiker, there is no [medical school] curriculum for poverty medicine:

No one teaches “The Art of Medical Decision Making With Limited Funds” or “Medical Compromise with Cultural Strictures”. Medical practice in a community of poor people often seems a solitary specialty without research, common cause, or shared-experience. I and my few partners are isolated professionally, with no way to even to assess our own record … As a physician for the poor, I know there will be no “professional advancement”. The bottom rung of the ladder is the same as the top rung: working as a clinic doctor, seeing patients day-to-day.” (quoted in *The Renewal of Generosity* by Arthur Frank [47]).

In addition, few studies related to care of patients with chronic pain include any discussion of gender or race. In an important study, Mendoza and her colleagues conducted an ethnographic case study of Staten Island, analyzing popular media press and interviews with physicians and pharmacists [48]. They note that, “Our analysis of the opioid epidemic in Staten Island illustrates how narratives of white victimization and contagion are sanitized, rationalized, and reproduced throughout the community and among health care providers.” They go on to state that the “narrative strategy of focusing on the dangers of substances themselves, rather than on the social conditions leading to widespread exposure to substances” represents a long-standing absence in the literature. Such insights underscore the importance of adopting a critical intersectionality lens in this area.

Low-income patients often are unable to access treatments such as physiotherapy, massage, and even mental health care that are frequently offered instead of opioids. As our participants state, more affluent people have access to these therapies through private insurance or have the means to pay out-of-pocket. Restricting access to these services to the wealthy is a joint outcome of what is a trend in health care reform. The Ontario health care system is increasingly characterized by economic-rationalist discourse, and thus by neoliberal reforms [49–51]. Key components of such reforms include faith in the freedom of the market, increasing privatization of all spheres of life, and promoting an ideology that privileges the empowered individual who takes active control of their “lifestyle” [52, 53]. As shown by a robust body of literature, such trends render health and well-being matters of personal rather than societal or government responsibility, while simultaneously depoliticizing it. As many have already explored, the move towards viewing health as a matter of personal responsibility implicitly advances the notion that the chronically ill are responsible for their difficult circumstances [50–53].

The physicians in our study provide numerous examples of the tension they feel between wanting to believe and trust their patients, being aware of their difficult life circumstances, and yet increasingly being directed to suggest alternative health therapies that are far beyond the reach of most patients. The subjective nature of pain also adds to the difficulties they have in believing their patients’ accounts. In their excellent ethnographic study of U.S. clinicians’ experience of contending with pain in their everyday practice, Crowley-Matoka and colleagues describe how “pain and pain medications remain both incompletely medicalized and ineffectively medicalizing in American biomedicine [54]”. Our findings support this characterization. Many of the clinicians in our study described being caught between losing access to traditional treatments such as pharmaceutical pain relief on the one hand and having to offer patients of lower socio-economic status individualized strategies of self-management (e.g. mediation, massage, physiotherapy, exercise) that are not covered by provincial health plans. As many of our research participants highlighted, in most cases these are both woefully inadequate for addressing patients’ pain in and of themselves, and are also unfeasible given the broader challenges that these patients face.

In our analysis we traced how care is standardized through textually established policies that must be enacted across multiple locations. Recent undertakings enacted jointly by the CPSO and the Ontario Ministry of Health in an effort to provide provincial oversight of opioid prescribing were frequently evident in interviews both directly and indirectly. There are a number of ways in which our research shows the harmful effects of this. Examples such as physicians who are dismissive of patients suggest that such ideologies are present in Ontario system and may be increasing due to the pressure on physicians to police their patients who suffer from chronic pain, ironically to avoid being policed themselves by their professional College. This leads to physician dissatisfaction and in many cases distress as their work shifts away from diagnosis and healing.

It also leaves both physicians and patients troubled by the inadequate care that is being provided and a growing sense of helplessness as physicians are left to manage rising social inequities without support or training [55], the symptoms of which may be masked in the language of the “opioid crisis” in the field of chronic pain. While we do not dispute that there is an epidemic of harm and mortality associated with opioid use, we would cast this as a public health issue rather than a strictly medical crisis. The notion of an opioid crisis discursively locates the problem in a class of drugs rather than in institutional priorities and historical processes in which it is embedded. In addition, as we have noted elsewhere, escalating concerns about so-called drug-seeking patients reflects a situation “in which blame for the institutionalized lack of support and treatment is transferred to the most vulnerable actors in the social organization of care” [56]. Situating the problem as a public health issue, however, allows for solutions such as harm reduction that are practiced by communities, have strong physician support and yet are currently being contested across Canada [57].

## Strengths and limitations

Institutional ethnography is a particular qualitative approach that allows researchers to link the everyday experiences of participants working lives to the wider institutional factors influencing that experience through an examination of texts including discourse. In our study we were able to link physician accounts of stress, emotional exhaustion and even depersonalization with the many examples they provided of treating patients with low SES, within the context of changing legislation by their professional Colleges. We noted how much of the work that physicians described was about gatekeeping or policing patient access to chronic pain medication rather than the work of healing. This puts the provider in a conflicted position relative to their patients in need as the institutional priority is restricting or withholding therapies once widely prescribed and now recognized as risky.

While our findings have profound implications for patient care in Ontario, we have not focused on patients and thus cannot authoritatively speak to how our findings may implicate them. Indeed, in this article their experiences as they are presented here are in most cases refracted through the voice of their physicians. Such perspectives are unlikely to reflect a comprehensive understanding of patient experience. Future research could be aimed at understanding the experiences of patients of low SES with chronic pain in order to better understand how their work is hooked into and organized by these same institutional priorities.

## Conclusion

We have drawn together discourses on health and social inequality and the rising literature on physician well-being. Indeed, the pervasiveness of poverty among chronic pain patients means that care providers are being asked to mediate across class lines, to help people whose problems they have not been trained to solve. This results in a lot of anguish and worry for them.

While the association between wealth and health has long been recognized, solutions for this are increasingly being sought through medicine, and in this process are frequently recast as medical problems–such as an opioid crisis–rather than as issues of public health. In addition, while opioid tapering is recommended as the antidote to this problem, and while sanctions are levelled against physicians who over-prescribe, there are currently few alternative therapies available that are affordable for many patients. We would argue that this represents a medicalization of poverty that diverts attention and resources from social services and education and toward clinical treatment where physicians are poorly situated to manage the situation. The provision of stronger supports, rather than potential sanction, would offer much needed assistance to those physicians dealing with the crisis of growing inequity on the front lines. In addition, if physicians are to taper or stop prescribing opioids for their patients, there must be fully funded alternatives for them to offer. Without this funding in place, the current regulations and guidelines risk estranging physicians from the very patients who most need their care and will contribute to both patient suffering and threaten physician well-being in the future.

## Supporting information

S1 File (DOC)

S2 File (DOC)

## References

[1] Goldberg DS, McGee SJ. Pain as a global public health priority. BMC public health. 2011 12;11(1):770. 21978149 10.1186/1471-2458-11-770 PMC3201926

[2] STATSCAN. Pain or discomfort that prevents activities. 2016. Available from: https://www150.statcan.gc.ca/n1/pub/82-229-x/2009001/status/pdl-eng.htm

[3] Pefoyo AJ, Bronskill SE, Gruneir A, Calzavara A, Thavorn K, Petrosyan Y, et al The increasing burden and complexity of multimorbidity. BMC Public Health. 2015 12;15(1):415. 25903064 10.1186/s12889-015-1733-2 PMC4415224

[4] Nicholson K. Examining multimorbidity in primary health care using a pan-Canadian electronic medical record database. Presented at the Trillium Primary health Care Research Day Conference; 2015; June 4; Toronto, ON.

[5] Hogan ME, Taddio A, Katz J, Shah V, Krahn M. Incremental health care costs for chronic pain in Ontario, Canada: a population-based matched cohort study of adolescents and adults using administrative data. Pain. 2016 8 1;157(8):1626–33. 10.1097/j.pain.0000000000000561 26989805

[6] Lynch ME. The need for a Canadian pain strategy. Pain Research and Management. 2011;16(2):77–80. 21499581 10.1155/2011/654651 PMC3084407

[7] Todd KH, Ducharme J, Choiniere M, Crandall CS, Fosnocht DE, Homel P, et al Pain in the emergency department: results of the pain and emergency medicine initiative (PEMI) multicenter study. The journal of pain. 2007 6 1;8(6):460–6. 10.1016/j.jpain.2006.12.005 17306626

[8] Cragg J, Warner FM, Shupler MS, Jutzeler CR, Cashman N, Whitehurst DGT, et al Prevalence of chronic pain among individuals with neurological conditions. STATSCAN; 2018 Available: https://www150.statcan.gc.ca/n1/pub/82-003-x/2018003/article/54921-eng.htm] 29561565

[9] Work Wellness and Disability Prevention Institute. What is Chronic Pain? 2018 Available: https://www.wwdpi.org/ChronicDisease/WhatIsChronicPain

[10] Tang NK, Crane C. Suicidality in chronic pain: a review of the prevalence, risk factors and psychological links. Psychological medicine. 2006 5;36(5):575–86. 10.1017/S0033291705006859 16420727

[11] Canadian Institute for Health information. Opioid crisis having “significant” impact on Canada’s health care system. 2018 Available: https://www.cihi.ca/en/opioid-crisis-having-significant-impact-on-canadas-health-care-system

[12] King NB, Fraser V, Boikos C, Richardson R, Harper S. Determinants of increased opioid-related mortality in the United States and Canada, 1990–2013: a systematic review. American journal of public health. 2014 8;104(8):e32–42. 10.2105/AJPH.2014.301966 24922138 PMC4103240

[13] Smolina K, Gladstone E, Morgan SG. Determinants of trends in prescription opioid use in British Columbia, Canada, 2005–2013. Pharmacoepidemiology and drug safety. 2016 5;25(5):553–9. 10.1002/pds.3989 26947145

[14] Marshall JR, Gassner SF, Anderson CL, Cooper RJ, Lotfipour S, Chakravarthy B. Socioeconomic and geographical disparities in prescription and illicit opioid-related overdose deaths in Orange County, California, from 2010–2014. Substance abuse. 2018 4 4:1–7. 10.1080/08897077.2018.1442899 29465301

[15] Fitzpatrick T, Rosella LC, Calzavara A, Petch J, Pinto AD, Manson H, et al Looking beyond income and education: socioeconomic status gradients among future high-cost users of health care. American journal of preventive medicine. 2015 8 1;49(2):161–71. 10.1016/j.amepre.2015.02.018 25960393

[16] Rosella LC, Fitzpatrick T, Wodchis WP, Calzavara A, Manson H, Goel V. High-cost health care users in Ontario, Canada: demographic, socio-economic, and health status characteristics. BMC health services research. 2014 Dec;14(1):532. 25359294 10.1186/s12913-014-0532-2 PMC4221677

[17] Marmot M, Allen J, Bell R, Bloomer E, Goldblatt P. WHO European review of social determinants of health and the health divide. The Lancet. 2012 9 15;380(9846):1011–29. 10.1016/S0140-6736(12)61228-8 22964159

[18] Raphael D. Addressing health inequalities in Canada. Leadership in Health Services. 2002 6 1;15(2):1–8.

[19] Jouini G, Choinière M, Martin E, Perreault S, Berbiche D, Lussier D, et al Pharmacotherapeutic management of chronic noncancer pain in primary care: lessons for pharmacists. Journal of pain research. 2014;7:163 10.2147/JPR.S56884 24711711 PMC3969347

[20] Choinière M, Dion D, Peng P, Banner R, Barton PM, Boulanger A, et al The Canadian STOP-PAIN project–Part 1: Who are the patients on the waitlists of multidisciplinary pain treatment facilities?. Canadian Journal of Anesthesia/Journal canadien d'anesthésie. 2010 6 1;57(6):539–48. 10.1007/s12630-010-9305-5 20393821

[21] Smith D. Institutional Ethnography In: May T, editor. Qualitative research in action. Sage; 2002 P. 2–13.

[22] Webster F, Bhattacharyya O, Davis D, Glazier R, Katz J, Krueger P, et al An Institutional Ethnography of Chronic Pain Management in Family Medicine (COPE) Study Protocol. *BMC Health Services Research*. 2015 9 8;5(9):e007664. 10.1186/s12913-015-1078-7 PMC4634802 26541288

[23] Smith DE, editor. Institutional ethnography as practice. Rowman & Littlefield; 2006.

[24] Smith DE. Institutional ethnography: A sociology for people. Toronto: Rowman Altamira; 2005 6 3.

[25] DeVault ML, McCoy L. Institutional ethnography: Using interviews to investigate ruling relations Institutional ethnography as practice. Lanham, MD: Rowman & Littlefield; 2006 p. 15–44.

[26] Campbell M, Gregor F. Mapping Social Relations: A primer in doing institutional ethnography. Garamond Press; 2002.

[27] O'Reilly K. Ethnographic methods. London: Routledge; 2012.

[28] Webster Fiona, Rice Kathleen. Conducting ethnography in primary care. *Journal of Family Practice*, forthcoming 5 2019. 10.1093/fampra/cmz061 PMC6859508 31617894

[29] Busse JW, Craigie S, Juurlink DN, Buckley DN, Wang L, Couban RJ, et al Guideline for opioid therapy and chronic noncancer pain. Canadian Medical Association Journal. 2017 5 8;189(18):E659–66. 10.1503/cmaj.170363 28483845 PMC5422149

[30] C, Jackson SE. The measurement of experienced burnout. Journal of organizational behavior. 1981 4;2(2):99–113.

[31] Canadian College of Physicians and Surgeons. CPSO Opioid Strategy. n.d. Available: https://www.cpso.on.ca/CPSO/media/documents/Positions%20and%20Initiatives/Opioids/Opioid-Fact-Sheets.pdf

[32] Olah ME, Gaisano G, Hwang SW. The effect of socioeconomic status on access to primary care: an audit study. Canadian Medical Association Journal. 2013 1 1:cmaj-121383. 10.1503/cmaj.121383 PMC3612171 23439620

[33] Franks P, Fiscella K. Effect of patient socioeconomic status on physician profiles for prevention, disease management, and diagnostic testing costs. Medical care. 2002 8 1:717–24. 10.1097/00005650-200208000-00011 12187185

[34] Wear D, Kuczewski MG. Perspective: medical students’ perceptions of the poor: what impact can medical education have?. Academic Medicine. 2008 7 1;83(7):639–45. 10.1097/ACM.0b013e3181782d67 18580079

[35] Simmenroth-Nayda A, Görlich Y. Medical school admission test: advantages for students whose parents are medical doctors?. BMC medical education. 2015 12;15(1):81. 25898946 10.1186/s12909-015-0354-x PMC4409754

[36] Puddey IB, Mercer A. Socio-economic predictors of performance in the Undergraduate Medicine and Health Sciences Admission Test (UMAT). BMC medical education. 2013 Dec;13(1):155. 24286571 10.1186/1472-6920-13-155 PMC4220554

[37] Dhalla IA, Kwong JC, Streiner DL, Baddour RE, Waddell AE, Johnson IL. Characteristics of first-year students in Canadian medical schools. Canadian Medical Association Journal. 2002 4 16;166(8):1029–35. 12002979 PMC100877

[38] Magnus S.A., Mick S.S. Medical schools, affirmative action, and the neglected role of social class. Am J Public Health. 2000;90:1197–1201 10936995 10.2105/ajph.90.8.1197 PMC1446350

[39] Van Ryn M, Burke J. The effect of patient race and socio-economic status on physicians' perceptions of patients. Social science & medicine. 2000 3 1;50(6):813–28. 10695979 10.1016/s0277-9536(99)00338-x

[40] Woo JK, Ghorayeb SH, Lee CK, Sangha H, Richter S. Effect of patient socioeconomic status on perceptions of first-and second-year medical students. Canadian Medical Association Journal. 2004 6 22;170(13):1915–9. 10.1503/cmaj.1031474 15210639 PMC421718

[41] Kennedy M, Kennedy M. Bogan Bias: Addressing Class-Based Prejudice in Physician-Patient Interactions. Journal of Social Inclusion. 2014 12 15;5(2):27–43.

[42] Raingruber B. (2014). Health disparities In Raingruber B. (Ed.), Contemporary health promotion in nursing practice (1st ed., pp. 123–160). Burlington, MA: Jones & Bartlett Learning.

[43] Major B, Mendes WB, Dovidio JF. Intergroup relations and health disparities: A social psychological perspective. Health Psychology. 2013 5;32(5):514 10.1037/a0030358 23646834 PMC3988903

[44] Street RL, Gordon H, Haidet P.Physicians' communication and perceptions of patients: Is it how they look, how they talk, or is it just the doctor? Social Science & Medicine. 2007; 65 (3): 586–598. 17462801 10.1016/j.socscimed.2007.03.036 PMC2811428

[45] Willems S, De Maesschalck S, Deveugele M, Derese A, De Maeseneer J. Socio-economic status of the patient and doctor–patient communication: does it make a difference?. Patient education and counseling. 2005 2 1;56(2):139–46. 10.1016/j.pec.2004.02.011 15653242

[46] Beagan BL. Everyday classism in medical school: Experiencing marginality and resistance. Medical Education. 2005 8;39(8):777–84. 10.1111/j.1365-2929.2005.02225.x 16048620

[47] Frank Arthur W. (2004). The Renewal of Generosity: Illness, Medicine, and How to Live. Chicago: University of Chicago Press.

[48] Mendoza S, Rivera AS, Hansen HB. Re-racialization of Addiction and the Redistribution of Blame in the White Opioid Epidemic. Med Anthropol Q. 2018 4 27. 10.1111/maq.12449 29700845

[49] Aronson J., & Sammon S. (2000). Practice amid Social Service Cuts and Restructuring: Working with the Contradictions of "Small Victories." Canadian Social Work Review/Revue canadienne de service social, 167–187.

[50] Aronson J., & Smith K. (2009). Managing restructured social services: Expanding the social? British Journal of Social Work, 40(2), 530–547

[51] Rankin J.M., & Campbell M. L. (2006). Managing to Nurse: Inside Canada’s Health Care Reform. Toronto: University of Toronto Press.

[52] Galvin R. Disturbing notions of chronic illness and individual responsibility: Towards a genealogy of morals. Health:. 2002 4;6(2):107–37.

[53] Rose N., Miller P., (2008). Governing the present: administering economic, social and personal life. Cambridge: Polity Press.

[54] Crowley-Matoka M., True G. (2012) No One Wants To Be the Candy Man: Ambivalent Medicalization and Clinician Subjectivity in Pain Management. Cultural Anthropology 27 (4): 689–712.

[55] Pinto AD, Bloch G. Framework for building primary care capacity to address the social determinants of health. Can Fam Physician. 2017 11;63(11):e476–e482. 29138172 PMC5685463

[56] Webster F, Bremner S, Oosenbrug E, Katz J, McCartney C. From opiophobia to overprescribing: A critical scoping review of medical education training for chronic pain. *Pain Medicine*. 2017 3 23. 10.1093/pm/pnw352 PMC5914373 28371881

[57] Goldberg DS, McGee SJ. Pain as a global public health priority. BMC public health. 2011 12;11(1):77 21978149 10.1186/1471-2458-11-770 PMC3201926

