# Supplementary material for: An ethnography of chronic pain management in primary care: The social organization of physicians’ work in the midst of the opioid crisis
Source: PLoS One. 2019 May 1;14(5):e0215148. doi: 10.1371/journal.pone.0215148 (PMC6493733; doi:10.1371/journal.pone.0215148)
Supplement: S1 File — (DOC) [file pone.0215148.s001.doc]

**Preamble**

Thank you for agreeing to participate in this interview. We are interviewing you to better understand your experiences of providing care to those patients you consider complex patients as well as those who have chronic OA pain. So, there are no right or wrong answers to any of our questions, we are interested in your expertise of the everyday work that you perform.

You have signed the consent form and are aware of your rights as a participant. The interview should take approximately 30 to 60 minutes depending on how much information you would like to share. With your permission, I would like to audio record the interview because I don’t want to miss any of your comments. All responses will be kept confidential. This means that your de-identified interview responses will only be shared with research team members and we will ensure that any information we include in our report does not identify you as the respondent. You may decline to answer any question or stop participating in the focus group at any time and for any reason. May I turn on the digital recorder?

____________________________________________________________________________

**General description of current practice (or work situation)**

I’m going to start by asking you to describe your current practice (please note that ***the specificities*** of this question will be modified to fit with the work, broadly defined, of the person being interviewed. The ***topic areas*** will remain similar).

Prompts: How long have you been in practice here? Who else is in your practice? Who do you communicate with on a daily basis? How do you mostly communicate with them (email, phone, in person)?

Can you take me through a typical day?

Prompts: What sorts of things do you do? How do you know to do that? How would you describe what you do if you were talking to someone (like me) who wasn’t a clinician?

**Descriptions of complex patients**

Before we begin, I want to ask you, what springs to mind when you hear the term “complex patient”?

How would you describe the typical complex patient to a resident?

Prompts: That’s an interesting term. Can you tell me a little bit more about that? What does it mean to you specifically? Do you recall when you first heard or read that term?

**Experiences of patients with chronic pain (or care for chronic pain)**

Can you tell me in a general way about your experiences of complex patients with chronic pain?

Now, can you give me a concrete example of a time when you provided care for such a patient and just walk me through what you did to provide care for them?

Prompts: What did you do? How did you know to do that? Did you have to fill out any forms? Was anyone else involved with that work?

When you’re doing the work you have just described, what are some of the main challenges you face? How do you manage them?

**Conclusion**

- Is there anything else that you would like to comment on in relation to this topic that you feel is important and that I might have left out?

Thank you very much for your time and the information you shared today.
