# Supplementary material for: An ethnography of chronic pain management in primary care: The social organization of physicians’ work in the midst of the opioid crisis
Source: PLoS One. 2019 May 1;14(5):e0215148. doi: 10.1371/journal.pone.0215148 (PMC6493733; doi:10.1371/journal.pone.0215148)
Supplement: S2 File — (DOC) [file pone.0215148.s002.doc]

**Guide for Observations**

The Research Associate (RA) conducting the interviews will be designated to observe across several clinical settings, including sitting in on patient appointments and to observe a variety of clinical tasks of the primary healthcare providers solicited for interviews. In keeping with the broader study, the purpose is to observe the everyday work of care for complex patients within local clinical sites while attending to how and through what social relations, policies and texts the work is coordinated with other settings. Important data to observe includes what language and discourse is invoked, what connections to other care settings are created (through referrals, recommendations, ordering tests, prescriptions, etc) and what forms are utilized. Consent will differ according to where observations are being conducted. For example, when observing in a larger pain clinic, prior to commencing, an email letter will be sent out by the appropriate head of the clinic to inform faculty, staff and residents of this project. When the RA is sitting in on patient-physician appointments the physician will first ask the patient for verbal consent, as is standard practice when introducing medical students and residents. The RA will be introduced transparently to patients and the study will be briefly described. In all situations verbal consent will be obtained by the RA from staff personnel at the beginning of all observations. She will also remain alert for non-verbal signs that her presence is intrusive and quietly withdraw from observations if this occurs. The RA will make field notes and memos, predominantly after the observed interactions are complete although some scratch notes might be taken during observations. The appointments will not be audio recorded and all information will be kept confidential, de-identified in any subsequent reports, and stored safely in a locked office and/or on password-protected computers in the office at the Department for Family and Community Medicine. Information noted about patients will be general, restricting to broad descriptions such as sex, age, and health status. Both the patient and provider have the right to object to the presence of the RA at any time or to specific information items being captured, and these procedures and rights will be explained to the patients upon introducing the RA and the study. Occasional ad hoc interviews with providers outside patient interactions will be recorded with the provider’s verbal and written consent. The RA will practice ongoing and continuous consent by checking in with providers at appropriate intervals to ensure that they are in agreement with the observations proceeding.

Our guide is based on Spradley’s nine dimensions of descriptive observation^1^:

The EVENT refers to the particular occasion that brings everyone together, in this case the clinical appointment. First, the observer will describe the SPACE (physical layout of the operating room) and the ACTORS (the people involved in the setting, identified by profession rather than name, ie family physician). She will also then record the nature of the ACTIVITIES of the various actors, including their GOALS, paying specific attention to communication as well as the OJBECTS being used or in the room. She will note individual actions that stand out (ACTS). She will record this information sequentially (TIME). She will also be mindful of her perception of the FEELINGS of the group (i.e. collegiality, frustration, anger,).

The observer will augment her growing understand of the issues surrounding communication in the clinical appointment by speaking with various ACTORS *after* the appointment (note that she will never speak to patients). She will seek verbal consent to ask questions. Her questions will take the form of the following series of questions: “I noted that [describe ACTIVITY] happened during the appointment. Can you please tell me your understanding of this? Is this typical?”. Or, “I noted that [ACTOR] seemed to be responsible for [GOAL]. Is this accurate? She will take care not to ask leading questions or to use names during these exchanges.

References

1. Spradley, James P. (1980). Participant Observation in Participant Observation*.* Orlando, Florida: Harcourt College Publishers.
2. Martyn Hammersley & Paul Atkinson (1983). Recording and organizing data in Ethnography. London: Routledge.
3. Atkinson, P. & Coffey, A. (2003). Revisiting the relationship between participant observation and interviewing. In J. Holstein & J. Gubrium (Eds.) Inside interviewing: New lenses, new concerns (pp415-427). Thousand Oaks: Sage.
4. Emerson, R., Fretz, R. & Shaw, L. (2001). Participant observation and fieldnotes. In P. Atkinson, A. Coffey, S. Delamont, J. Lofland & L. Lofland (Eds.) Handbook of ethnography (pp. 352-368). London; Sage.
5. Diamond, T. (2006). “Where did you get the fur coat, Fern?” Participant observation in Institutional Ethnography. In D. Smith (Ed.) Institutional Ethnography as Practice *(pp. 45-63)* Lanham, MD; Rowman & Littlefield.
